# Supplementary material for: Loss of Elp1 disrupts trigeminal ganglion neurodevelopment in a model of familial dysautonomia
Source: eLife. 2022 Jun 17;11:e71455. doi: 10.7554/eLife.71455 (PMC9273214; doi:10.7554/eLife.71455)
Supplement: Transparent reporting form [file elife-71455-transrepform1.pdf]

1st Floor
24 Hills Road
Cambridge CB2 1JP, UK

P 01223 855340
W elifesciences.org
T @elife

eLife's transparent reporting form

We encourage authors to provide detailed information *within their submission* to facilitate the interpretation and replication of experiments. Authors can upload supporting documentation to indicate the use of appropriate reporting guidelines for health-related research (see EQUATOR Network), life science research (see the BioSharing Information Resource), or the ARRIVE guidelines for reporting work involving animal research. Where applicable, authors should refer to any relevant reporting standards documents in this form.

If you have any questions, please consult our Journal Policies and/or contact us: editorial@elifesciences.org.

Sample-size estimation

- You should state whether an appropriate sample size was computed when the study was being designed
- You should state the statistical method of sample size computation and any required assumptions
- If no explicit power analysis was used, you should describe how you decided what sample (replicate) size (number) to use

Please outline where this information can be found within the submission (e.g., sections or figure legends), or explain why this information doesn't apply to your submission:

Samples sizes were determined based on previous publications that examined this mouse model or similar mouse models of Familial Dysautonomia, which generally used 2-4 embryos of each genotype to report a given phenotype, as the *Elp1 CKO* phenotypes are highly penetrant (Jackson, et al. 2014, *Development*, George et al. 2013 *PNAS*, Li et al. 2020, *JCI*). This information is stated in Methods under the "Animal Husbandry" section.

Replicates

- You should report how often each experiment was performed
- You should include a definition of biological versus technical replication
- The data obtained should be provided and sufficient information should be provided to indicate the number of independent biological and/or technical replicates
- If you encountered any outliers, you should describe how these were handled
- Criteria for exclusion/inclusion of data should be clearly stated
- High-throughput sequence data should be uploaded before submission, with a private link for reviewers provided (these are available from both GEO and ArrayExpress)

Please outline where this information can be found within the submission (e.g., sections or figure legends), or explain why this information doesn't apply to your submission:

A biological replicate is defined, in this work, as one embryo and each "N" listed throughout the manuscript refers to the number of biological replicates. Technical replicates only apply to Figure 7J, and are defined as serial horizontal sections through the trigeminal ganglion, i.e., there were 5 sections (technical replicates) counted per each embryo, and there were 3 embryos measured per each condition. Availability of experimental embryos was limited due to the Covid-19 pandemic and associated research/travel restrictions. Where possible we examined at least 3 embryos per condition per analysis, but occasionally we reported on a minimum of 2 embryos per condition due to these restrictions. We always examined embryos from at least 2 different litters within an analysis to account for variations that may occur within a single litter. We also included littermates as Controls to account for this variation. All "N"s are stated within the Methods and within appropriate Figure Legends.

Statistical reporting

- Statistical analysis methods should be described and justified
- Raw data should be presented in figures whenever informative to do so (typically when N per group is less than 10)
- For each experiment, you should identify the statistical tests used, exact values of N, definitions of center, methods of multiple test correction, and dispersion and precision measures (e.g., mean, median, SD, SEM, confidence intervals; and, for the major substantive results, a measure of effect size (e.g., Pearson's r, Cohen's d)
- Report exact p-values wherever possible alongside the summary statistics and 95% confidence intervals. These should be reported for all key questions and not only when the p-value is less than 0.05.

Please outline where this information can be found within the submission (e.g., sections or figure legends), or explain why this information doesn't apply to your submission:

Details of statistical analyses can be found in the Methods, as well as in the Figure Legends for each experiment.

(For large datasets, or papers with a very large number of statistical tests, you may upload a single table file with tests, Ns, etc., with reference to sections in the manuscript.)

Group allocation

- Indicate how samples were allocated into experimental groups (in the case of clinical studies, please specify allocation to treatment method); if randomization was used, please also state if restricted randomization was applied
- Indicate if masking was used during group allocation, data collection and/or data analysis

Please outline where this information can be found within the submission (e.g., sections or figure legends), or explain why this information doesn't apply to your submission:

Embryos were placed into respective groups (Control or *Elp1 CKO*) based on genotype. While the genotype of each embryo was determined before the time of analysis, the researcher was blinded during data collection to prevent bias. Details of which genotypes constitute Controls vs *Elp1 CKO*s can be found in the Methods under "Animal Husbandry".

Additional data files ("source data")

- We encourage you to upload relevant additional data files, such as numerical data that are represented as a graph in a figure, or as a summary table
- Where provided, these should be in the most useful format, and they can be uploaded as "Source data" files linked to a main figure or table
- Include model definition files including the full list of parameters used
- Include code used for data analysis (e.g., R, MatLab)
- Avoid stating that data files are "available upon request"

Please indicate the figures or tables for which source data files have been provided:

We have source data files uploaded for Figures 2-9.
